# Implementation of clinical guidelines on diabetes and hypertension in urban Mongolia: a qualitative study of primary care providers’ perspectives and experiences

**DOI:** 10.1186/s13012-015-0307-0

**Published:** 2015-08-11

**Authors:** Oyun Chimeddamba, Anna Peeters, Darshini Ayton, Enkhjargal Tumenjargal, Sonin Sodov, Catherine Joyce

**Affiliations:** Department of Epidemiology and Preventive Medicine, School of Public Health and Preventive Medicine, Monash University, 99 Commercial Road, Melbourne, VIC Australia 3004; Mongolian Association of Family Medicine Specialists, Street of Prime Minister Amar, Sukhbaatar District-1, Ulaanbaatar, 14210 Mongolia; Obesity and Population Health, Baker IDI Heart and Diabetes Institute, 99 Commercial Road, Melbourne, VIC Australia 3004; Department of Health Development, National Center of Public Health, Peace Avenue 17, Bayanzurkh District-3, Ulaanbaatar, Mongolia

## Abstract

**Background:**

Hypertension and diabetes, key risk factors for cardiovascular disease, are significant health problems globally. As cardiovascular disease is one of the leading causes of mortality in Mongolia since 2000, clinical guidelines on arterial hypertension and diabetes were developed and implemented in 2011. This paper explores the barriers and enablers influencing the implementation of these guidelines in the primary care setting.

**Methods:**

A phenomenological qualitative study with semi-structured interviews was conducted to explore the implementation of the diabetes and hypertension guidelines at the primary care level, as well as to gain insight into how practitioners view the usability and practicality of the guidelines. Ten family health centres were randomly chosen from a list of all the family health centres (*n* = 136) located in Ulaanbaatar City. In each centre, a focus group discussion with nurses (*n* = 20) and individual interviews with practice doctors (*n* = 10) and practice managers (*n* = 10) were conducted. Data was analysed using a thematic approach utilising the Theoretical Domains Framework.

**Results:**

The majority of the study participants reported being aware of the guidelines and that they had incorporated them into their daily practice. They also reported having attended guideline training sessions which were focused on practice skill development. The majority of participants expressed satisfaction with the wide range of resources that had been supplied to them by the Mongolian Government to assist with the implementation of the guidelines. The resources, supplied from 2011 onwards, included screening devices, equipment for blood tests, medications and educational materials. Other enablers were the participants’ commitment and passion for guideline implementation and their belief in the simplicity and practicality of the guidelines. Primary care providers reported a number of challenges in implementing the guidelines, including frustration caused by increased workload and long waiting times, time constraints, difficulties with conflicting tasks and low patient health literacy.

**Conclusions:**

This study provides evidence that comprehensive and rigorous dissemination and implementation strategies increase the likelihood of successful implementation of new guidelines in low resource primary care settings. It also offers some key lessons that might be carefully considered when other evidence-based clinical guidelines are to be put into effect in low resource settings and elsewhere.

## Background

Hypertension and diabetes, key risk factors for cardiovascular disease, are significant health problems globally. Over 80 % of the 17 million cardiovascular disease (CVD) deaths in 2008 occurred in low- and middle-income countries [1]. Evidence indicates that the risk of cardiovascular disease and diabetes can be reduced through the adoption of a healthy diet, regular physical activity and avoidance of tobacco use [2]. In Mongolia, CVD and cancers have become the leading causes of mortality since 2000 [3, 4]. In 2013 the prevalence of hypertension was 27.5 % and diabetes 8.3 % [5]. A recent study found that only 17 % of the total population with hypertension and 26 % of people with diabetes in Mongolia reported receiving lifestyle modification interventions (LMIs) [6].

Primary health care has been the central strategy in expanding access to a range of preventive services in many low- and middle income countries [7]. A family group practice system in Mongolia was established under the name ‘family clinics’ in the early 2000s with the assistance of the First Health Sector Development Program funded by the Asian Development Bank (ADB). The family clinics were renamed as ‘family health centres’ (FHCs) according to the Health Law amended in 2011 and are private entities funded to service communities by the Government with services being free of charge to residents of Mongolia [8–12]. FHCs are the major facilitators of primary health care in Mongolia, and are operated as team-based private group practices. Of the 228 FHCs nationwide, 136 are located in Ulaanbaatar City (the capital of Mongolia) covering a population of 1.3 million people and the remainder (92) are in provincial centres of rural areas serving populations of more than half million people [3].

Evidence-based clinical guidelines for assessing risk factors for noncommunicable diseases (NCDs) are an important component of prevention interventions in response to the NCD epidemic [13]. In 2011, the working group of the Ministry of Health (MOH) Mongolia in co-operation with Millennium Challenge Account-Mongolia (MCA-M) Health Project developed and published clinical guidelines on arterial hypertension and diabetes (hereafter referred to as the guidelines) [14]. The MOH working group included representatives from the Mongolian Heart Association, Health Sciences University of Mongolia, Mongolian Cardiologists’ Association, Mongolian Electrocardiographists’ Association, Mongolian Family Doctors’ Association, private hospitals, Pharmacy School and School of Nursing, and the National Institute for Health and Welfare (THL) and EPOS Health Management team in Mongolia. The content and recommendations of the guidelines were developed based on the most recent international guidelines on the management of hypertension and diabetes such as the World Health Organization (WHO) and the European and Finnish national guidelines on the management of hypertension and diabetes. Although the guidelines covered a broad range of screening and management aspects of hypertension and diabetes, the guidelines take particular account of lifestyle interventions [15–17]. The main target was the screening of persons aged between 40 and 64 years. This involved taking body measurements, testing for a random or fasting blood glucose and cholesterol, assessing risk scores and provision of advice regarding LMIs (Table 1).

**Table 1:** Lifestyle modification interventions recommended by the guidelines in Mongolia [14]

Intervention types	Targets
Salt intake	Less than 6 g salt/day = 2400 mg sodium
Fruit and vegetables	Increase to 5 servings per day
Saturated (animal) fat	Replace butter with vegetable oil or margarine
	Use low fat or skimmed milk products
	Reduce the use of fatty meat products
	Eat fish 1–2 times weekly
Fibres	Increase the use of whole grain products, fruits and vegetables
Alcohol drinking	Abstain from drinking alcohol. If not possible, decrease the amount of alcohol consumed to less than 2 standard drinks^a^ daily for men and less than 1 standard drink daily for women. Avoid binge drinking
Overweight and obesity	Weight reduction. The recommended body mass index is 18.5–25 kg/m2. The recommended waist circumference is <90 cm in men and <80 cm in women
Physical activity	At least 30 min at a time ≥5 occasions in a week
Smoking	Cessation

^a^One standard drink, small bottle of beer (330 ml), one glass wine (150 ml), 50 ml of vodka

However, guidelines uptake is often dependent on a number of factors including the effectiveness of clinical guideline implementation strategies [18, 19]. A systematic review of the effectiveness of clinical guideline implementation strategies, predominantly in developed countries, found that comprehensive, proactive, face-to-face and participatory learning environments improved implementation. By contrast, postal delivery of guidelines or web-based distribution of guidelines with no further implementation strategies were regarded as passive and ineffective strategies [20]. Prior to this study, there was no assessment of how the guidelines have been implemented across the primary care level in Mongolia and little in other low resource settings.

This study aimed to explore the implementation of the guidelines with an emphasis on LMIs from the perspective of primary care providers. We sought to identify factors that primary care providers believed supported the implementation of these guidelines in urban Mongolia.

## Methods

### Design and sampling

A phenomenological qualitative study to explore implementation of the guideline recommended LMIs was employed. The research was conducted in Ulaanbaatar City, Mongolia. Ulaanbaatar, the capital city of Mongolia, was chosen as 68.1 % of the total population reside there, and because its population has been growing rapidly due to internal migration, socio-economic development, enhanced employment opportunity and improved living conditions [3]. An initial step of sampling frame was a list of all the FHCs located (*n* = 136) in Ulaanbaatar City. We used the public domain of the Mongolian Association of Family Medicine Specialists as a sampling frame. A preliminary letter about the study was emailed in advance to all FHCs informing potential participants that they may soon be randomly selected for the study. Qualitative studies often use purposive sampling methods, which are based on theoretically relevant characteristics that guide selection of participants. However, in our study, we used random sampling to select ten FHCs from the sampling frame. This was based on our interest in investigating typical experiences of implementing the guidelines, and the fact that there were no pre-determined theoretical considerations to indicate that a purposive approach would be preferable. Once ten FHCs had been randomly selected, the researcher contacted heads of the selected FHCs explaining the purpose of the research and asked permission for their staff to take part in the study. As the heads of the selected ten FHCs agreed, doctors and nurses were invited to participate in the study. All ten selected FHCs and their staff consented to participate in the study. In this study, we experienced a 100 % participation rate from the contacted centres. This is likely due to the top-down management approach in team-based primary care facilities and the cooperative nature of manner of service providers as a part of cultural background of medical professionals in Mongolia. Monetary incentives equivalent to their average daily wage offered to recompense for their time and contribution in the study, as they were interviewed during working hours. Ethical approvals for the study were obtained from the Monash University Human Research Ethics Committee and the Ministry of Health (Mongolia) Ethics Committee. All participants gave informed consent.

### Participants and data collection

To capture diversity in implementation experiences and practices, study participants were recruited from managers of the FHCs, practice doctors and nurses. A total of 40 primary care providers from ten general practices in urban Mongolia participated in the study. Data were collected between November 2013 and February 2014. Ten managers and ten practice doctors participated in semi-structured individual interviews to explore their opinions, experiences, understanding and motivations in the context of the guideline implementation [21]. Twenty primary care nurses participated in focus groups with two to three participants in each (ten focus groups in total). A semi-structured interview guide was developed and modified slightly to tailor to the different participant groups. Prior to data collection, two public health officers in Mongolia reviewed the interview guide to check whether the questions were worded clearly and appropriately. Focus groups and individual interviews lasted for 60 to 90 min. The interviews were arranged at a convenient time for the participants and were conducted with face to face in a meeting room at their practice. The research study was conducted in the local language by OC who is native Mongolian, is experienced in interviews and focus group discussions with health professionals and familiar with the health care system setting in Mongolia.

We adopted the Theoretical Domains Framework (TDF), which includes 14 domains as listed in Table 2, to guide data collection and analysis [22–24]. This set of domains has been applied previously in a wide range of clinical guidelines to understand the determinants of key barriers and enablers to implementation practice as perceived by health professionals [25, 26]. The TDF is a series of behaviour change conceptual determinants and associated constructs arising from psychological and organisational theory [22]. Implementation researchers highlighted that these constructs may influence behaviours of service providers as enablers or barriers. Guidelines normally require change in clinical practice to provide evidence-based care so that better health outcomes for patients are achieved. Therefore, the TDF was considered to be an appropriate framework for this study. All interviews and discussions were audio-recorded, transcribed verbatim and translated into English. Researcher field notes were retained.

**Table 2:** Theoretical domains and constructs of the TDF and the corresponding questions for interviews

Domains and their constructs	Interview prompts
Knowledge: - Knowledge about the guidelines - Knowledge about LMIs of the guidelines - Knowledge sources	• Are you aware of the guidelines on hypertension and diabetes? Can you please explain a little? • When and how did you receive the guidelines? • Where do you obtain information on the CVD and diabetes prevention?
Skills: - Clinical skills - Counselling skills/the lifestyle modification component of the guidelines - Frequently recommended LMIs	• How did you learn how to advise the LMIs? • Did you have any training on the guideline recommendations? • Which LMIs are provided as part of daily routine practices?
Beliefs about capabilities: - Technical capacity - Professional competence - Empowerment - Self-esteem - Belief in outcomes of the guidelines - Belief in clinical practice change	• What things help you to implement the lifestyle modification interventions effectively in your centre? • In what ways have the guidelines been useful in your FHC for the prevention of diabetes and CVD? • How did the guidelines change your practice?
Beliefs about consequences: - Challenges in the guideline implementation - External evaluation and review - Internal evaluation and review	• What do you see are the difficulties in implementing the prevention recommendations in your practice? • Tell me about the specific examples of the challenges that you have faced? • What kind of monitoring tools or systems do you use to keep track of how the lifestyle interventions are being offered at your centre?
Perceptions of roles and responsibilities: - Roles and responsibilities - Organisational support - Teamwork - Hierarchal pressure - Supervision - Feedback - Community support - Partnership	• What do you see as your role in the CVD and diabetes prevention? • What are the roles and responsibilities of the various staff in implementing the guidelines? • Do some staff have greater capability in this than others? • Can you describe any community linkages your FHC has been engaging with in order to effectively deliver the LMIs of the guidelines?
Environmental context and resources: - Resources/material/technology - Environmental barriers	• What support have you been receiving to implement the prevention recommendations of the guidelines over time? • What support is available/ are you aware of any support? • What do you see are the difficulties in implementing the prevention recommendations in your practice?
Leadership/optimism: - Organisational commitment - Individual commitment	• Emerged after participants had been interviewed
Reinforcement: - Implementation procedure - Actual clinical change - Developing alternative strategies - Barriers and enablers - Field visits - Monitoring system	• What kind of monitoring tools or systems do you use to keep track of how the LMIs are being offered at your centre?
Motivation/goals: - Goal setting - Target setting - Future priority	• What makes the lifestyle interventions like these successful in your practice? • What practical steps would be beneficial to enhance implementation of the lifestyle modification interventions?
Memory, attention and decision processes: - Decision-making	• In what circumstances do you refer to these guidelines?
Behavioural regulation	• Are there processes in your FHC that encourage the use of the guidelines? • What makes the lifestyle interventions like these successful in your practice?
Nature of the behaviours	• Do you practise the lifestyle modification component/prevention of the guidelines? • What procedures do you practise to intervene the lifestyle modifications? • What do you advise your clients on CVD and diabetes?

### Analysis

The main analysis strategy was thematic analysis [27]. Interviews were transcribed and imported into NVivo 10 for data management and analysis [28]. OC conducted the interviews, read the transcripts two to three times and developed a coding guide using a process of deductive coding based on the interview schedule and the domains of the TDF. The first round of coding involved the development of these deductive codes. The second round of coding was inductive, and OC identified and confirmed emerging themes through open, axial and thematic coding [29, 30]. Transcripts and emerging themes were circulated to AP and DA and discussed for verification.

## Results

Key findings for each of the domains and constructs of the TDF are presented below: knowledge, skills, belief about capabilities, belief about consequences, social/professional role, environmental context and resources, leadership and optimism, reinforcement and barriers to the guideline implementation.

### Knowledge

All participants were asked if they were aware of the guidelines and the specific aspects of recommendations such as the LMIs. The majority of participants were aware of the guidelines. Many participants mentioned the guideline launching events that were held in late 2011 by the MOH and MCA-M Health Project. During this launch, hard copies of the guidelines were distributed. A series of training sessions was also identified as a key enabling factor by primary care providers as they felt professionally and intellectually prepared for the guideline arrival. The training was recalled as having been both theoretically oriented and practical.

…The training I attended gave me great evidence that offering lifestyle intervention is a key in the prevention of cardiovascular disease and diabetes. Before the training I did not know that giving advice is the main task we need to convey in prevention (Practice doctor, FHC3).

### Skills

Most participants noted that they had learnt new skills including clinical, counselling and communication skills. For example, they felt that their skills in explaining to patients the causes and consequences of being overweight and obese, teaching the calculation of body mass index and providing advice about diet and physical activity had improved.

…If my patient is overweight or obese, I advise to eat less, avoid oily foods and sweets, exercise more by running and walking actively at least for a half an hour a day so that they get sweaty. If someone is mildly hypertensive, I advise them not to become too stressed, to be relaxed, and to manage their weight according to their height and eat more green vegetables (Practice doctor, FHC8).

### Beliefs about capabilities

Most participants believed in their capacity and professional competence to deliver the guideline recommendations, and that this was due to the extensive training sessions and a continuous supply of medical devices and hard copy distribution of the guidelines and educational materials. In contrast, a few nurses occasionally reported marginally dissenting views that they were less confident in interpreting borderline test results and providing specific lifestyle recommendations to patients. They related this lack of confidence to the fact that they did not have opportunities to attend training.

…Frankly speaking, I did not know much about diabetes before the guidelines came into place; my knowledge was very general, and narrow. I used to refer suspected cases or cases with clear symptoms to the district health centre for further investigation; I did not deal with such cases myself. I used to approach diabetic cases in a very unprofessional manner—but now things have changed (Practice doctor, FHC6).

### Perceptions of roles and responsibilities

#### Organisational support

Neither the guideline developers nor the MOH gave instruction on roles and responsibilities. Nevertheless, most practice managers of FHCs perceived themselves as being active stakeholders in the delivery of the guideline recommendations. In this regard, many practice managers advised that they had designed action plans under which practice doctors and nurses had been instructed to be responsible for certain tasks. Nurses were commonly given responsibility for performing body measurements, risk score assessments, tests for blood glucose and cholesterol, conducting educational activities and advising on general LMIs. Practice doctors were normally responsible for making clinical decisions and providing patients with detailed description of the LMIs.

#### Teamwork

Most participants valued the importance of teamwork. Many practice doctors and nurses appreciated a leadership role of their practice managers having led organisational re-structuring by creating nursing and quality monitoring teams. They believed teamwork contributed to ensure continuous quality improvement in the processes of the guideline implementation and increased professional confidence in primary care practice.

…One of my roles is running short training sessions for patients and offering the lifestyle change advice. Although I am responsible for the noncommunicable disease program, not everything is dependent on me. We work as a team. Other providers also examine patients, detect cases, measure body measurements, test for sugar and cholesterol and offer the lifestyle change advice towards the prevention of hypertension and diabetes (Nurse, FHC7).

### Environmental context and resources

#### Technical support/pre-implementation support

The major enabler to the delivery of the LMIs recommended by the guidelines as perceived by all study participants was the technical support provided by the Government of Mongolia jointly with the MCA-M Health Project. The introduction of the guidelines was accompanied by the provision of a number of necessary basic medical appliances such as devices to measure height, weight and waist circumference; equipment to measure blood glucose, cholesterol and blood pressure; and stethoscopes. Most participants reported using the distributed supplies to implement the guidelines.

…All the supplies given to us were not for a single time, they have been supplied over and over again. All those supplies are still available for us to use when we screen, when we test, when we examine our patients. This was just amazing support! (Practice doctor, FHC5).

A large quantity of training manuals, handouts, posters and written materials on NCD prevention were reported as having been distributed several times to both health professionals and the general public.

### Leadership

The creation of teams to implement guidelines was initiated and led by practice managers of the FHCs in all cases. Most of them reported being prepared to take over the necessary management and leadership role by creating teamwork environments and designing a new organisational structure to fit into a new practice, upgrading their human resources and involving specialists and communities in meeting client demands. During the implementation, several practice managers reported having hired social workers and psychologists, aiming to focus on individual counselling on a variety of sensitive and personal aspects of the LMIs. They reported feeling passionate, committed and recognised that ‘we must implement this program’, which was a reflection of high organisational commitment.

…The implementation of the guidelines depended on us. Doctors and nurses should participate equally in implementing the guidelines because it requires teamwork and ongoing effort (Practice manager, FHC1).

### Enthusiasm/optimism

Most participants acknowledged that the guidelines articulated all procedures step by step and tied individual activities into a coherent system. All participants felt that the guidelines were certainly implementable, easy to understand, simply written, compact and explicit.

…We were all very committed and enthusiastic to implement the guidelines. This was a completely new approach to primary care services. In other words, this was a new delivery system design. The guidelines were the first ever explicit and practical tool from a public health perspective (Practice doctor, FHC10).

An interesting finding emerged during this study: Many primary care providers stressed that although they have been facing a number of challenges in the delivery of the guideline recommendations, they did not regard them as major problems. Instead, they focussed on the fact that they had an important role in the guideline implementation, felt accountable and responsible for following the recommended procedures and recognised the importance of NCD prevention.

### Reinforcement and monitoring of guideline implementation

Amongst the FHCs in this study, diverse monitoring approaches were applied. A number of FHC practice managers reported that they monitored through random observations how doctors and nurses offered the lifestyle interventions and followed up with group discussions on areas for improvement. A few participants reported that a cross-professional appraisal, monitoring and evaluation tool had recently been approved by Ministerial decree and introduced nationwide. With the use of this tool, a doctor monitors a doctor and a nurse monitors a nurse. The tool involves an appraisal of whether blood pressure is measured correctly, patient information is recorded fully and lifestyle modification advice is offered. The findings are discussed between providers themselves. The majority of participants advised that monitoring groups from the MOH and district health department often conducted field visits to FHCs to monitor the implementation process of the guidelines and provided feedback to improve their performance.

### Barriers to the guideline implementation

#### Time

One of the leading barriers identified to the delivery of the LMIs recommended by the guidelines was a lack of time that was consistent across all participants. Asking necessary questions to get an understanding of what dietary preferences and concerns patients had and engaging in productive conversation with the patient were perceived by many participants as essential for achieving mutually acceptable health decisions. This often resulted in long waiting time for other patients, which was identified as the next barrier. In Mongolia, there is a strong culture under which patients come to FHCs without having made a prior appointment.

…When I examine my patient by measuring height, weight, waist circumference and offer some advice on the lifestyle modification interventions, it takes a lot of time. The people waiting outside often get anxious and complain about why it is taking such a long time, why doctors are just chatting and they even complain that they will report this to the MOH (Practice doctor, FHC1).

#### Increased workload

Another major barrier identified by most study participants was their increased workload resulting from clinical consultations and procedures recommended by the guidelines. A number of practice doctors and nurses admitted that they were unable to fully cover all aspects of the guideline recommendations with every patient.

…I offer patients lifestyle change recommendations based on body measurements and blood test results. It takes much time. When there is high workload that makes it more difficult to offer advice fully. I can’t stick with one patient for more than 10–15 min as many patients are waiting outside and some of cases require urgent care (Practice doctor, FHC9)

#### Patient preferences and beliefs

Many study participants emphasised that although they offered the lifestyle change modifications to those at high risk of developing diabetes and CVD, some patients preferred to take medications rather than adhere to the lifestyle interventions.

…I had a case…when I offered a man with high blood pressure, the lifestyle modification recommendations and sent him home. But what he did was, he was seen by another doctor at our centre and got medicine. That means some people believe that just medicines and pills will quickly decrease their blood pressure, they do not regard the lifestyles they are engaged in as a trigger for disease onset (Practice doctor, FHC2).

#### Patient health literacy

Another barrier identified was the low level of patient health literacy. Some study participants, especially practice doctors and nurses, pointed out that because some patients have no basic understanding on the importance of the lifestyle on disease onset, much time is spent in explaining all the basic health information. It appears that a lack of health literacy adversely affects decisions made by patients in relation to following a healthy lifestyle.

…One other difficult matter was delivering of health messages. When we are explaining about the prevention of diabetes, patients did not understand the point that we wanted to convey, so we were explaining one thing over and over again (Nurse, FHC5).

#### Patient socio-economic status

Some participants expressed the opinion that people from less affluent backgrounds were far less likely to adhere to the suggested lifestyle change modifications because they were unable to afford the more costly healthy foods.

…It is hard for some people to comply with our lifestyle advice, especially for low income households, as sometimes they don’t even have enough for their basic needs. So they are less likely to prioritise their health. They usually say that ‘we do not have enough money so how could we buy things you suggest’ (Nurse, FHC6).

## Discussion

This qualitative study is the first to explore the perceptions of staff of the implementation practices of the diabetes and hypertension guidelines with special emphasis on the LMIs at the primary care level in urban Mongolia. Using the TDF, our study has identified a number of enablers and barriers to the uptake of the guidelines and, in particular, regarding the delivery of lifestyle changes recommended in the guidelines, as perceived by the participants. The key enablers reported were provision of extensive training courses to introduce the guidelines and their components, delivery of a substantial volume of published guidelines and supply of a considerable amount of screening equipment. On the other hand, the potential barriers discovered included a lack of time, increased workload, competing task pressure, patients’ preferences and beliefs and their low health literacy.

The TDF domains ‘knowledge’, ‘skills’, ‘beliefs about capabilities’, ‘perceptions of roles and responsibilities’, ‘leadership’ and ‘environmental context and resources’ were most frequently identified. The majority of domains related to enablers rather than barriers to the implementation practices of the guidelines. Nevertheless, the thematic analysis underlined the importance of relationships between domains and the implementation practices. The domains ‘knowledge’ and ‘skills’ appeared to be the drivers of the guideline implementation and relationships with other domains. Although the domain ‘motivation and goals’ was identified infrequently, primary care providers felt motivated to practice new guidelines as their knowledge and skills were improved following a series of training courses. Moreover, improved knowledge and skills appeared to impact the providers’ ‘motivation and goals’, ‘commitment’ and ‘beliefs about capabilities’.

The TDF domains ‘knowledge’, ‘skills’ and ‘beliefs about capabilities’ reflected in the following attributes. Research evidence suggests that application of the new clinical guidelines in general practice is largely achieved in steps: First, there is a process of ensuring that doctors and nurses are fully trained and educated in the guidelines. Then, acceptance of the practice change occurs [31, 32]. The findings from the present study indicate that these steps were carefully thought through and considered in the implementation of the guidelines in the Mongolian setting. For example, primary care providers accepted the recommendations of the guidelines and felt that these were important activities for preventing diabetes and CVD. Consequently, a number of novel clinical procedures became routine, such as body measurements, testing for blood glucose and cholesterol, risk score assessment and provision of the LMIs. Similar changes have been observed in numerous research studies, for example, in the Netherlands [31–33] and in Korea [34]. Here, we provide similar evidence for a low resource setting. Conversely, there is research evidence that many service providers still do not follow the key recommendations in guidelines [25, 35–37]. In these research studies, a lack of knowledge, inaccessibility and unavailability of the guidelines and a lack of support and enthusiasm for them were reported as the main obstacles to their implementation, and there was also a lack of follow up monitoring by the Government (or governing body) of specific implementation strategies. The importance of those strategies is confirmed both by research findings showing the negative impact that occurs when these are lacking and our study indicating the positive impact occurring when they are present.

The domain ‘perceptions of roles and responsibilities’ appeared to be unique to the guideline implementation. Although research suggests that service providers should be responsible for guideline implementation, that responsibility can be varied according to authority, power and funding [38]. Notably, the cultural context of the medical profession in Mongolia and the structure of the health care system and funding mechanism are relatively distinct from those in many Western and European countries. The health care system in Mongolia is characterised by top-down (vertical) management, and primary care facilities are entirely funded by the state budget. By contrast, primary care in many Western and European countries is private enterprises. Although in many cases, they receive government funding or subsidies, they operate as autonomous private businesses. The Code of Ethics of Medical Professionals defines a set of guiding principles for professional conduct throughout the health sector of the country [39]. It is to be followed by every medical professional and every organisation in the sector. Consequently, once the guidelines were approved officially, providers felt that they were morally and professionally accountable for the guideline implementation. This suggests that there are cultural and legal backgrounds attached to primary care that providers should consider when seeking to implement newly introduced guidelines. Grol argues that implementing guidelines may be achieved by obligations, rules and laws albeit their continuing effect remains uncertain [31]. In many other countries, the autonomy of doctors and general practice clinics is such that this top-down approach that applies in Mongolia may not be relevant despite similar underlying principles applied. This demonstrates that the medical professional culture in Mongolia is less focused on professional autonomy than in other places.

The domain ‘leadership’ offered positive illustration that most FHC practice managers took the leadership role in the process of the guideline implementation by creating a teamwork environment, providing managerial and peer support, designing a new organisational structure, upgrading human resources and involving specialists and communities. All these factors, including organisational commitment and individual optimism and enthusiasm, facilitated uptake of the guidelines. These findings are consistent with other studies that report that facilitating a supportive environment, developing collaborative relationships, maintaining a positive attitude and promoting learning about chronic diseases are effective strategies that may improve adherence to newly recommended clinical practice [40–42].

The domain ‘environmental context and resources’ was identified as enabler to primary care providers to practice the guideline implementation, in particular, formal training courses, availability of the guidelines and provision of equipment and resources. A series of interactive training sessions provided to service providers at the primary care level appeared to be the key drivers for gaining systematic knowledge and practice skill development to be able to deliver the guideline recommendations. In addition, distribution of a wide range of resources that had been supplied to them by the Mongolian Government complimented to assist with the implementation of the guidelines. The resources, supplied from 2011 onwards, included screening devices, equipment for blood tests, medications and educational materials. Other enablers comprised of monitoring of progress, audits of practice, provision of feedback, support from managers, commitment and passion, belief in the simplicity and practicality of the guidelines. Therefore, investing in implementation strategies that go beyond passive dissemination of guidelines is required for improved implementation. This is supported by the findings from research undertaken by Grol [31, 33], Grimshaw et al. [43] and others [44].

The domain ‘environmental context and resources’ was also identified as barrier to primary care providers that limit their ability to perform the recommendations due to environmental factors [45]. In our study, the majority of the study participants complained that their performance was affected by the following barriers, such as time constraints, demands from other competing tasks and increased workload, that prevent them from adhering to the recommendation of the LMIs, and these barriers remain as large barriers for primary care providers. Amongst them, a lack of time was the most reported barrier to the effective delivery of the LMIs to patients. This was consistent with a number of other similar studies and therefore should be carefully considered prior to the launching of further clinical guidelines [42, 46, 47]. We urge that the issuing of guidelines should be preceded by workload studies as well as situational analyses so that barriers to the implementation are minimised. The clash between the excessive waiting for patients and the lack of available time for doctors could be addressed by implementing more structured procedures for the giving of specific appointment times to individual patients. As an appointment system has been recently implemented in Mongolia, in particular, for secondary and tertiary care levels, it could be looked at as a potential in the primary care setting. A number of primary care providers in this study that identified patient preferences for medication were potential barriers inhibiting adherence to the LMIs recommended in the guidelines. The providers perceived that patients assumed that taking medicine is easier, simpler and faster than taking part in lifestyle changes. This thinking is associated with low level of their health literacy. This finding was consistent with qualitative research that reports the most salient influences on compliance that are the patients’ own beliefs, their knowledge, ideas and experiences [48]. Therefore, consideration to develop practical guides, for example, shopping and cooking guidebooks for different groups of the population, is recommended to support adherence to the guidelines.

### Limitations

The participating FHCs were randomly selected from an urban location and, therefore, may not represent rural FHCs’ experiences of guideline implementation. Also, the practice doctors and primary care nurses within the study FHCs were selected due to their willingness and availability to participate in the study. Hence, it is possible that those who chose to participate in the study are those who are more enthusiastic and committed to the guideline implementation. Consequently, those who have changed their clinical practice less in response to the guidelines would probably be less likely to take part in the study and this could result in overestimation of the extent of uptake of the guidelines. However, a large majority of those available were interviewed for the study. Lastly, it is worth noting that the reported benefits for patients and possible changes in their knowledge, attitude and skills are based on the participating primary care providers’ reports and that no direct measurement of patient outcomes or behaviours was done as part of this study.

This study was carried out in an urban city of Mongolia, and therefore, implementation processes may differ in rural or remote areas. A number of international studies suggest that those living in rural and remote areas experience poorer access to primary health care and poorer health outcomes compared with those living in metropolitan populations [49]. In particular, the statistics for diabetes show significant differences between metropolitan and rural areas [50]. Rates of death by diabetes in rural areas are notably higher than those experienced in metropolitan areas, and the diabetes differential is growing [50]. Consequently, it is possible that approaches to prevention of chronic illness may be dissimilar in rural areas compared with those in urban areas. Therefore, future research will be needed in order to enhance our understanding of approaches to prevention of noncommunicable disease in rural areas.

It is possible that participants in our study responded in ways which they perceived matched the expectations of the researchers (social desirability bias). For example, participants may have overstated the extent to which they were implementing the guidelines in practice. If this is the case, our study will overestimate the uptake and use of the guidelines. There are a number of features of our study which mitigate against this however. First, the sample of participating FHCs were randomly selected (with a 100 % response rate), not self-selected. Second, the interviewer assured the participants that ‘there are no right or wrong answers’ to reduce any concerns they might have about being judged for what they expressed. Furthermore, our primary focus was not on estimating the prevalence of uptake and use of the guidelines, but on describing the manner in which this was done.

## Conclusion

Approximately 3 years on from the publication of the first diabetes and hypertension guidelines in Mongolia, this study highlights the importance of investing comprehensively in strategies for implementation, without which any new guideline is less likely to be implemented successfully. The implementation strategy covers continuing medical education, face-to-face instruction, monitoring and feedback, supply and resource and ongoing support. It provides a number of key lessons, namely, barriers to be carefully considered when other evidence-based clinical guidelines are to be implemented in Mongolia and elsewhere. Mongolia, a low-income country with the double burden of disease, can serve as an example for other countries in the region and globally in implementation of evidence-based practice to achieve better health outcomes for patients. While participants in our study reported using the guidelines on a daily basis, the extent to which this reflects the level of adoption of the guidelines across all practices is unknown. The fact that FHCs in our study were randomly selected gives some confidence, further research specifically focused on uptake is required to confirm these findings.

## Abbreviations

ADB: Asian Development Bank
CVD: cardiovascular disease
FHC: family health centre
Guidelines: clinical guidelines on arterial hypertension and diabetes
LMIs: lifestyle modification interventions
MCA-M: Millennium Challenge Account-Mongolia
MOH: Ministry of Health
NCD: noncommunicable disease
TDF: Theoretical Domains Framework
WHO: World Health Organization
